# Survey of medical student attitudes regarding uterine transplant for cisgender and transgender women: an observational study

**DOI:** 10.1186/s12909-025-07588-8

**Published:** 2025-07-06

**Authors:** Brandon J. Kim, Deidre Hurse, Abram Brummett

**Affiliations:** Department of Foundational Medical Studies, Oakland University William Beaumont School of Medicine Oakland University, Rochester, Michigan, United States of America

**Keywords:** Uterine transplant, Cisgender, Transgender, Conscientious objection

## Abstract

**Background:**

This study wishes to survey medical students’ attitudes regarding legality of, funding for, and conscientious objection to uterine transplant (UTx) in cisgender and transgender women.

**Methods:**

Medical students were invited to complete an online anonymous survey from March 18, 2024 to April 1, 2024. Baseline demographics collected, and four-point Likert scales were used on four pairs of questions to evaluate attitudes regarding UTx for cisgender and transgender women. Subject responses to paired questions were analyzed using Fisher’s Exact Test. Strength of correlation between the paired questions were analyzed with Spearman’s Correlation.

**Results:**

A total of 96 responses were collected and 66 responses answering at least one of questions 5 to 8 were included in the final dataset. Nineteen (29%) self-identified as male and forty-three (65%) as female. A majority of respondents (72%) believed that a clinician should be able to object conscientiously to UTx regardless of gender identity, but only 14% would personally object to any UTx. A minority of respondents (10%) would object only to UTx for transgender women. Overall, female respondents (Correlation Coefficient average = 0.939) were more likely to select the same answer to paired questions regardless of gender identity compared to their male counterparts (Correlation Coefficient average = 0.558).

**Conclusions:**

This study shows some division among medical students regarding their attitudes toward UTx for cisgender and transgender women. For medical students willing to participate in UTx for cisgender but not transgender patients, additional ethical analysis is needed to determine whether these attitudes constitute invidious discrimination.

**Supplementary Information:**

The online version contains supplementary material available at 10.1186/s12909-025-07588-8.

## Background

The first uterine transplant (UTx) in the United States occurred in 2016 at the Cleveland Clinic and has become an effective treatment modality for women with absolute uterine factor infertility (AUFI) to gestate and deliver children. Multiple centers in the US are now offering UTx, and there is evidence that shows that the success rate is comparative with other infertility treatments [1]. Currently there exists the 2013 Montreal Criteria (ethical standard for uterine transplant) that requires recipients to be genetically female [2]. However, there has been animal model research showing that uterine transplant could be possible in the future for transgender women who were assigned male at birth [3]. One study showed that 94% of transgender women said the ability to gestate and deliver would be beneficial in their identity as women, and 88% expected a UTx to improve satisfaction with their gender identity [4]. 

If UTx becomes more commonplace in the future, it could benefit transgender men as well. 80% of transgender men with hysterectomy stated that they felt relief post-surgery, and 88% would have donated their uterus for transplant [5]. These hysterectomies could increase the number of organs available for living donor UTx.

With this advent of research there have been concurrent discussions of the ethics of UTx in transgender women [6]. Some concerns have been raised regarding needing robust safety data, maximizing benefit-risk ratios, and ensuring equal access after evidence of clinical viability [7]. Other ethical discussions and concerns have emerged about UTx and its implications, including procreative liberty, the right to gestate, and the expression of feminine identity in both cisgender and transgender women [8]. A recent scoping review of current literature discussing the ethical considerations of UTx in recipients other than cisgender women found 32 published articles, nearly half of which argued that transgender women are women who have the right to gestation. Justice was the most discussed topic, exploring concerns of discrimination in access, the legal definition of parenthood, and insurance coverage [9]. Another study in Turkey suggested that female physicians tend to have a more positive attitude towards transgender people, which can impact the care transgender patients receive [10]. In this study, we surveyed medical students at a single-campus medical school on their thoughts about research, funding, and conscientious objection to uterine transplantation for cisgender and transgender women.

## Methods

### Participants

Students actively enrolled at Oakland University William Beaumont School of Medicine (OUWB) in Rochester, MI, during the 2023–2024 academic year were eligible participants of the study, conducted from March 18, 2024 to April 1, 2024. Students are subject to a standard MD curriculum, with the first 2 years of study in basic sciences and the last 2 years of study in experiential clinical rotations in medical specialties. Of note, all OUWB students undergo longitudinal ethics courses in every year of their medical education.

### Survey instrument and administration

A de novo online survey instrument was created to identify attitudes. The survey was distributed to potential respondents via a current student E-mail listserv and private class-specific group chats. One reminder email was sent to the student E-mail listserv one week after the initial survey distribution. Responses were collected for 3 weeks in March and April 2024. After respondents were provided an introductory background to uterine transplants, age, gender, political affiliation, religious affiliation, ethnicity, and medical specialty interest data were collected. Four-point Likert scales were used on four pairs of questions to evaluate attitudes regarding UTx for cisgender and transgender women. In Question 5 respondents were asked whether they agreed or disagreed with whether UTx should be legal in the United States for cisgender and transgender women. In Question 6 respondents were asked whether they agreed or disagreed with whether government funding should be used to support research on UTx for cisgender and transgender women if it is legalized. In Question 7 respondents were asked whether they agreed or disagreed with whether clinicians should be able to conscientiously object to providing or assisting in UTX for cisgender and transgender women if it is legalized and clinically viable. In Question 8 respondents were asked whether they agreed or disagreed with whether they would conscientiously object to providing or assisting in UTX for cisgender and transgender women if it is legalized, clinically viable, and clinicians are permitted to conscientiously object. After each pair of questions, respondents were given the option to explain their reasoning in a free response format. Complete survey instrument questions are provided in the Supplementary Appendix SA1.

### Statistical analysis

Survey data were gathered and compiled using Qualtrics (Provo, Utah), an electronic survey platform. This process was conducted anonymously. Data were analyzed overall, and how subjects responded to questions 5 to 8 regarding cisgender women and transgender women. Continuous variables were presented as medians and interquartile ranges, whereas categorical variables are displayed as frequencies and percentages. To analyze how subjects responded to the two different aspects of the questions, Fisher’s Exact Test was utilized due to the small expected cell counts. To further investigate the strength of correlation between the questions, Spearman’s Correlation was used, given the ordinal nature of the data. All analyses were two-sided using an alpha of 0.05. Analyses were performed using SAS v9.4. The qualitative data collected through the optional free response format were not officially analyzed due to sample sizes smaller than the Likert-scale answers. For each paired question, three qualitative responses were chosen to display a variety of interesting opinions from different paired Likert-scale response combinations.

## Results

### Survey cohort demographics

A total of 96 responses were initially collected. Responses were included in the final analysis if participants answered at least one of questions 5 to 8, yielding a final sample size of 66. Of these, 63 respondents (95% of the analyzed sample) completed all questions on the survey. 19 (29%) self-identified as male and 43 (65%) self-identified as female. Of note, no respondents identified as transgender male or transgender female. All participants’ demographics are shown in Table 1.

**Table 1:** Demographics of the participants (*N* = 66)

**Age**	
N	66
Median (IQR)	25.0 (24.0, 26.0)
Mean (SD)	25.0 (1.75)
**Gender**	N (%)
Male	19 (28.8%)
Female	43 (65.2%)
Non-binary/Third-gender	1 (1.5%)
Prefer not to say	2 (3.0%)
Write-in	1 (1.5%)
**Political Affiliation**	N (%)
Republican	2 (3.0%)
Independent	10 (15.2%)
Democrat	29 (43.9%)
No affiliation	24 (36.4%)
Other	1 (1.5%)
**Religious Affiliation**	N (%)
Protestantism	11 (16.7%)
Catholicism	7 (10.6%)
Other Christian	10 (15.2%)
Judaism	3 (4.5%)
Islam	6 (9.1%)
Buddhism	2 (3.0%)
No affiliation	25 (37.9%)
Other	2 (3.0%)
**Ethnicity**	N (%)
African American or Black	3 (4.5%)
Asian	17 (25.8%)
Caucasian or White	39 (59.1%)
Latin or Hispanic	2 (3.0%)
Middle Eastern or Northern African Descent	5 (7.6%)
Other	2 (3.0%)
**Medical Specialty Interest**	N (%)
Anesthesiology	4 (6.8%)
Dermatology	1 (1.7%)
Emergency Medicine	4 (6.8%)
Family Medicine	2 (3.4%)
Gastroenterology	1 (1.7%)
Internal Medicine	12 (20.3%)
Medicine-Pediatrics	1 (1.7%)
Neurology	3 (5.1%)
Obstetrics & Gynecology	6 (10.2%)
Oncology	1 (1.7%)
Ophthalmology	1 (1.7%)
Orthopedic Surgery	3 (5.1%)
Pathology	1 (1.7%)
Pediatrics	6 (10.2%)
Physical Medicine & Rehabilitation	1 (1.7%)
Psychiatry	3 (5.1%)
Radiology	3 (5.1%)
Surgery	4(6.8%)
Urology	2 (3.4%)
Missing	7

Notes: IQR: interquartile range, SD: standard deviation

### Legality of UTx– Question 5

The survey results indicate substantial support for the legalization of UTx in the United States, with differing levels of support for cisgender and transgender women. For cisgender women, 90.9% of respondents (*N* = 60) either agree or strongly agree with legalization. In contrast, for transgender women, the support is lower, with 74.2% of respondents (*N* = 49) agreeing or strongly agreeing. A minority of respondents showed opposition to legalizing UTx for cisgender women, totaling 9.1% (*N* = 6), while for transgender women, this increased to 25.8% (*N* = 17). Of note, 16.7% of respondents (*N* = 11) agreed or strongly agreed with legalization of UTx for cisgender women but disagreed or strongly disagreed with legalization of UTx for transgender women. The survey reveals a noticeable variation in acceptance levels of UTx based on the demographic group, reflecting a broader acceptance for cisgender women compared to transgender women. The correlation coefficient (CC) for respondents’ answers regarding cisgender and transgender women was 0.697 (*p* <.0001), indicating a strong positive correlation and consistent responses. Results were statistically significant, with a p-value of less than 0.0001, suggesting a less than 0.01% chance of random variation. 47 respondents (71%) submitted free response answers. A full picture of paired responses is shown on Table 2. A selection of free response answers is shown on Table 3.

**Table 2:**
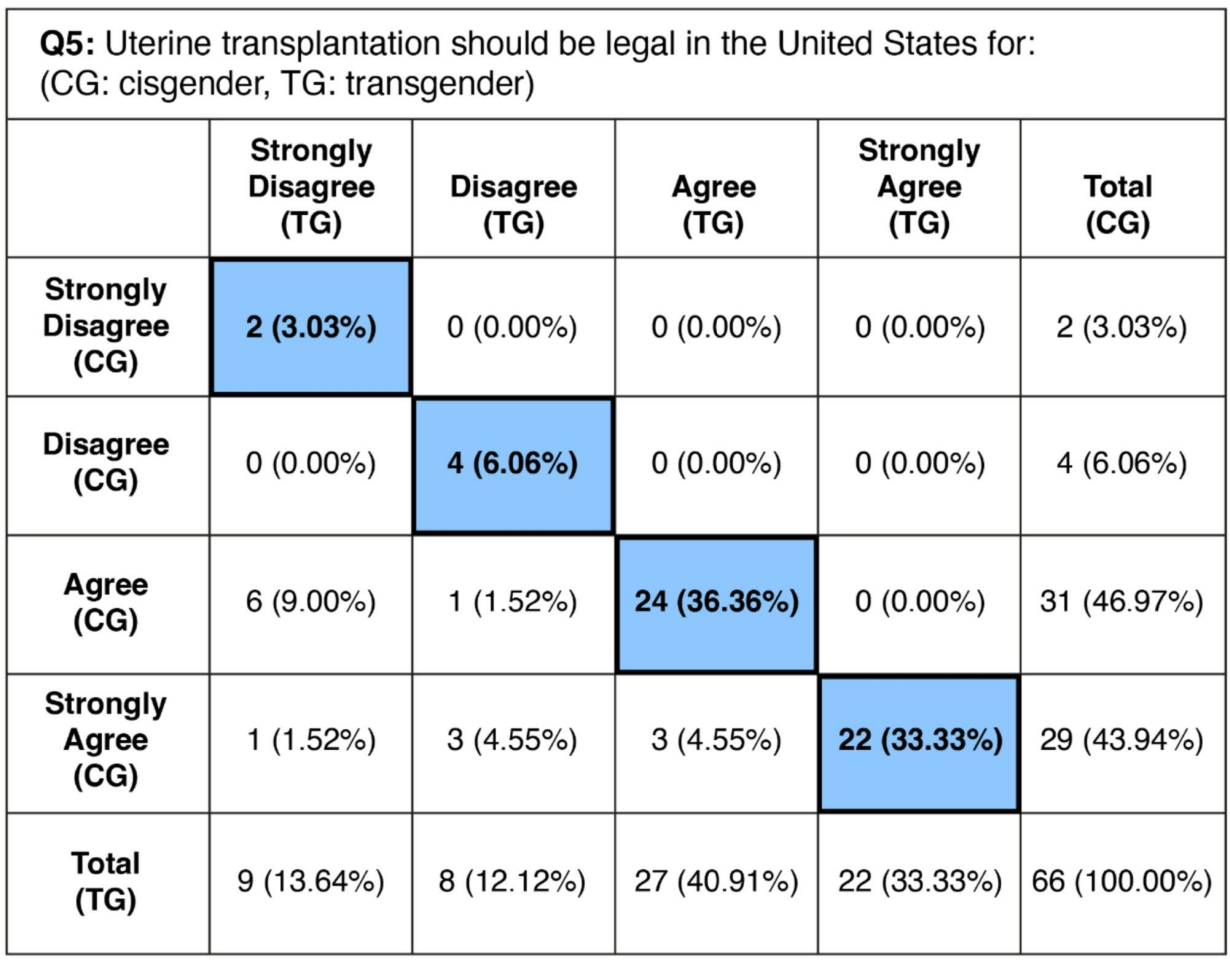
Frequency of each paired response type from survey question 5. Blue: frequency and percent of respondents choosing the same Likert-scale response for cisgender and transgender

**Table 3:** Select examples of free response answers with their likert scale answers from survey question 5

Likert scale answer to Question 5	Free response answer
Cisgender = Strongly Agree, Transgender = Strongly Disagree	“Biological men should not have uterine transplants. This extends farther than “identifying” with a gender and at this point has become associating “womanhood” with possession of a uterus. It is a desire to fulfill biological roles of female adults and as akin to an amputation identity disorder than a desire to be recognised as a woman.”
Cisgender = Agree, Transgender = Strongly Disagree	“It is my belief that biological females should be eligible for [uterine transplant]. Although the process seems incredibly challenging and poses a high risk of harm with the multiple surgeries and medications taken. It is my belief that adoption at that point would be the most beneficial. However, I do see why some would like to birth their own children rather than adopt. When it comes to transgender women receiving uterine transplants, I am not in favor of that. It strongly violates my religious views and is a violation of the God given bodies we possess.”
Cisgender = Agree, Transgender = Agree	“I am not sure I understand the objectionability of uterine transplantation for a transgender woman, from a medical perspective, if the ultimate result is delivery via C-section.…I do think that there are highly objectionable components of transgender care up to and including procedures like this, but as it stands right now I don’t think it is reasonable to eliminate this for transgender women.”

### Government funding on UTx research– Question 6

The survey showed strong support for government-funded research on uterine transplantation among both demographic groups. 83.1% of respondents(*N* = 54) agreed or strongly agreed that government funding should be used to support research on UTx for cisgender women, while 72.3% (*N* = 47) supported the same for research on UTx for transgender women. This indicates a difference in acceptance of funding for uterine transplantation research based on the targeted demographic group. Additionally, a small but significant fraction of respondents (16.9% for cisgender women and 27.7% for transgender women) disagreed or strongly disagreed with the funding. Of note, 10.8% of respondents (*N* = 7) agreed or strongly agreed with funding for cisgender women but disagreed or strongly disagreed with funding for transgender women. The CC for all respondents was 0.754 (*p* <.0001), indicating a robust positive correlation and high statistical significance. 35 respondents (54%) submitted free response answers. A full picture of paired responses is shown on Table 4. A selection of free response answers is shown on Table 5.

**Table 4:**
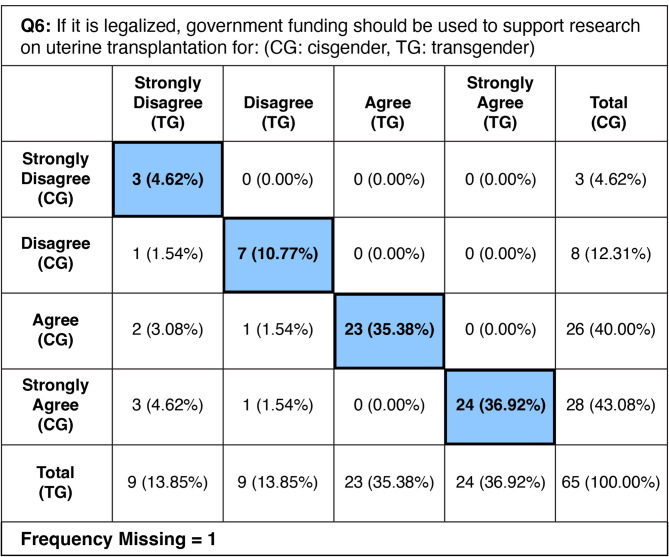
Frequency of each paired response type from survey question 6. Blue: frequency and percent of respondents choosing the same Likert-scale response for cisgender and transgender

**Table 5:** Select examples of free response answers with their likert scale answers from survey question 6

Likert scale answer to Question 6	Free response answer
Cisgender = Strongly Disagree, Transgender = Strongly Disagree	“Government funding should largely be allocated to life sustaining procedures, public health and social health functions.”
Cisgender = Strongly Agree, Transgender = Strongly Disagree	“It would be similar to requesting government funding for individuals who wish to remove their legs as it would make them feel complete.”
Cisgender = Strongly Agree, Transgender = Strongly Agree	“Transgender individuals are an underserved population that are subject to discrimination on a multitude of levels. The government should take action to reduce bias against underserved populations”

### Ability for clinicians to conscientiously object to UTx– Question 7

A majority of the respondents supported clinicians’ ability to conscientiously object to uterine transplantation (UTx) if it is legal and feasible for both cisgender and transgender women. 75.0% (*n* = 48) agreed or strongly agreed that clinicians should be able to object for cisgender women, while 76.6% (*n* = 49) supported the same for transgender women. This indicates widespread support for conscientious objection across both demographic groups. There was a notable alignment in opinions among the respondents. All 15 respondents (22.7%) who disagreed or strongly disagreed with conscientious objection for cisgender women also provided the same response for transgender women. Additionally, one respondent uniquely chose “disagree” for cisgender women and “agree” for transgender women, indicating a slight variation in individual responses. Overall, the data reflect a strong level of support for conscientious objection to UTx, with only a minority of respondents expressing opposition. This suggests a general acceptance of the concept regardless of the patient’s gender identity. The CC for all respondents was 0.961 (*p* <.0001), indicating a strong positive correlation and high statistical significance. 38 respondents (59%) submitted free response answers. A full picture of paired responses is shown on Table 6. A selection of free response answers is shown on Table 7.

**Table 6:**
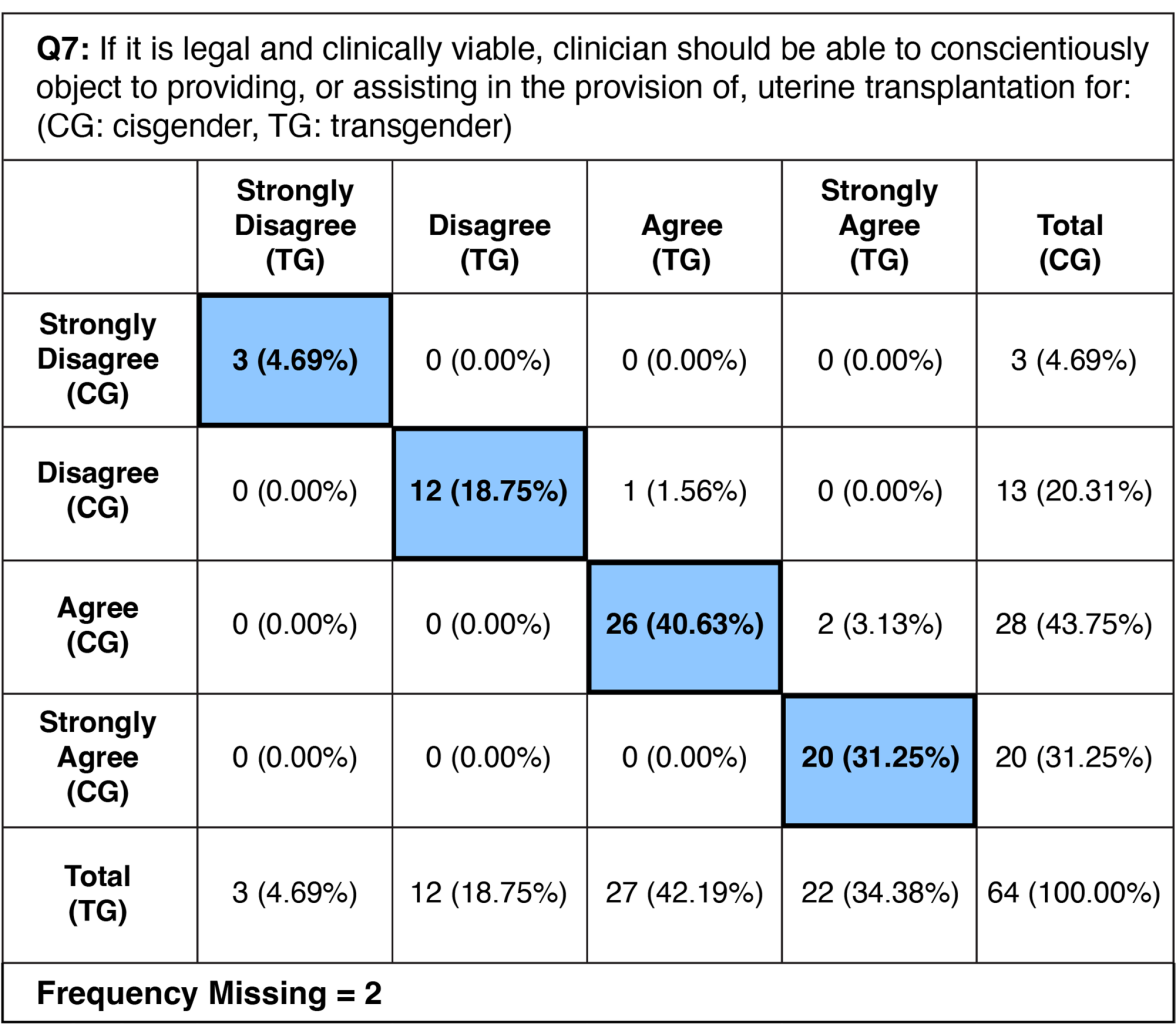
Frequency of each paired response type from survey question 7. Blue: frequency and percent of respondents choosing the same Likert-scale response for cisgender and transgender

**Table 7:** Select examples of free response answers with their likert scale answers from survey question 7

Likert scale answer to Question 7	Free response answer
Cisgender = Agree, Transgender = Agree	“This doesn’t seem like a procedure that would ever need to be done in an emergency situation, and I think that as long as there is no emergent or life threatening medical concern, conscientious objection WITH referral to another provider should be allowed.”
Cisgender = Disagree, Transgender = Disagree	“I can’t think of any beliefs that this ethically goes against, but if there is a religious or moral value that a lot of physicians hold preventing them from effectively and neutrally providing this care, then I would maybe switch to agree.”
Cisgender = Agree, Transgender = Agree	“While I strongly disagree with many of the reasons behind conscientious objections, I still think that clinicians should be allowed to exercise this right for some optional procedures.”

### Willingness to conscientiously object to UTx– Question 8

A majority of respondents indicated that they would not conscientiously object to providing or assisting in UTx if it is legal and clinically viable. Specifically, 85.7% (*n* = 54) of respondents selected “disagree” or “strongly disagree” for cisgender women, while 76.2% (*n* = 48) of respondents selected “disagree” or “strongly disagree” for transgender women. All 54 respondents who disagreed or strongly disagreed answered that they would not conscientiously object regardless of gender identity. Conversely, among those who selected “agree” or “strongly agree,” a total of 21.8% (*n* = 15) provided consistent answers for both cisgender and transgender women, with some selecting “agree” (*n* = 5 for cisgender, *n* = 6 for transgender) and “strongly agree” (*n* = 4 for cisgender, *n* = 9 for transgender). Notably, 9.5% (*n* = 6) of respondents chose a combination of “disagree/strongly disagree” for cisgender women and “agree/strongly agree” for transgender women. There were no respondents who selected the reverse combination. Overall, the data show a significant majority of respondents are against objecting to providing or assisting in UTx for both cisgender and transgender women, with a minority expressing a differing view depending on the patient’s gender identity. The CC for all respondents was 0.899 (*p* <.0001), indicating a very strong positive correlation and high statistical significance. 30 respondents (48%) submitted free response answers. A full picture of paired responses is shown on Table 8. A selection of free response answers is shown on Table 9.

**Table 8:**
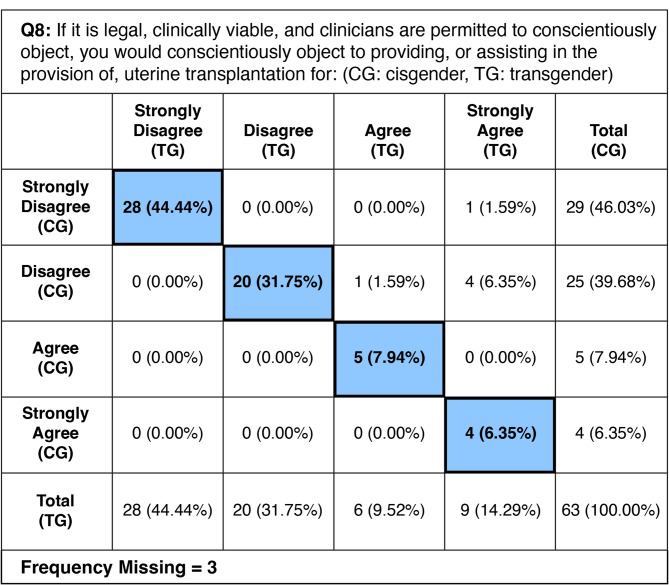
Frequency of each paired response type from survey question 8. Blue: frequency and percent of respondents choosing the same Likert-scale response for cisgender and transgender

**Table 9:** Select examples of free response answers with their likert scale answers from survey question 8

Likert scale answer to Question 8	Free response answer
Cisgender = Strongly Agree, Transgender = Strongly Agree	“If criteria is not met as outlined in previous responses for usage in cisgender women then I would due to its non-essential and largely invasive nature. Would object to all provisions for transgender women.”
Cisgender = Strongly Disagree, Transgender = Strongly Disagree	“If this procedure is legal, known to be effective, and I have the skill set to do it well and safely, then I see no reason to object.”
Cisgender = Strongly Disagree, Transgender = Strongly Agree)	“Correcting an organ defect is different than providing a service”

### Gender differences

CC analysis was performed for respondents who identified as male (*N* = 19, 28.8%) and female (*N* = 43, 65.2%). In all four paired questions, analysis revealed higher consistency in answers from female respondents (CC average = 0.939) compared to male respondents (CC avg = 0.558). This shows that female respondents demonstrated a higher likelihood of selecting similar answers compared to male respondents when asked about their attitudes regarding UTx in cisgender women vs. transgender women.

## Discussion

### Main findings

This study offers important insights into the attitudes of medical students at OUWB regarding UTx for both cisgender and transgender women. Most medical students shared similar opinions about UTx. However, a significant minority expressed differing views, with the essence of their perspectives indicating a preference for UTx for cisgender women over transgender women on issues of legality, funding, and the role for conscientious objection. This difference in opinion could be due to concerns about clinical feasibility, political and religious perspectives, or overall attitudes toward organ transplantation, which were some rationales mentioned by study participants. This division likely heralds challenges integrating UTx into future clinical practice.

### Interpretation

Given the ongoing legal debates surrounding gender-affirming treatments for minors, UTx for adult transgender patients may face similar legislative challenges [11]. Future physicians will likely play a crucial role in these debates. Open-ended responses from the survey highlighted several concerns, including doubts about the clinical viability and safety of UTx, as well as religious objections specifically targeting transgender UTx. One respondent raised a critical ethical question about associating “womanhood” with the possession of a uterus, challenging the notion of what defines a woman’s identity.

The issue of government funding and insurance coverage for transgender women seeking UTx raises ethical considerations [12]. The survey specifically asked about government funding for UTx research due to the procedure’s lack of clinical viability for transgender patients. Responses varied with some participants supporting UTx for transgender patients, others only supporting research for psychiatric support. One respondent opposed funding entirely, comparing it to government funding for procedures like healthy limb removal for patients with Body Identity Integrity Disorder [13]. Proponents of government funding cited benefits such as increased knowledge and equity of access, while opponents prioritized funding for other preventative or life-saving treatments.

Most respondents agreed that clinicians should have the right to conscientiously object to UTx, regardless of the patient’s gender identity. However, the significant minority who would object expressly to UTx for transgender patients but not for cisgender patients underscores the need for further ethical evaluation. This issue revolves around whether UTx for cisgender and transgender patients constitutes one procedure (transplantation of a uterus) or two (removing pathology for cisgender women or inducing pathology for transgender women) [14]. Inducing pathology is contrasted with preventing, curing, or mitigating pathologies, and is thought to be in conflict with the conception of the physician as healer. Whether physicians are ethically permitted to induce pathologies to achieve a broader sense of patient well-being is what gives rise to the conflict between pathocentric (the goal of medicine is health) vs. salutogenic (the goal of medicine is well-being) views that motivate many of the most contentious issues in medical ethics [15]. Each view of the goals of medicine will lead to a different conclusion as to whether conscientious objection in this context constitutes unfair discrimination [16]. While students plan to enter various specialties, many of which could involve UTx, even a single objecting clinician can create significant barriers to access.

Gender differences were also evident in the survey responses, with male respondents more likely to express different views on UTx based on gender identity than female respondents. Future research with larger sample sizes could explore other contributing factors, such as political or religious affiliations, that may explain these gender differences. More attitudinal research should be conducted once UTx is available to transgender women with known risks and benefits.

### Strengths and limitations

This study has some limitations. The survey was limited to students enrolled at OUWB during the study period, and the small sample size necessitates cautious interpretation of gender differences within this population. Although the survey was anonymous, there exists a possibility of social desirability bias, and some respondents may have felt pressure to align with what they perceive as the school’s values regarding transgender individuals. Future research should aim to survey a broader group of medical students across different medical schools and explore their attitudes toward uterus donation from cisgender women or transgender men with elective hysterectomies.

## Conclusions

The aim of this survey was to investigate the attitudes of medical students at OUWB regarding UTx for cisgender and transgender women. The results show that there is a wide range of opinions and attitudes toward UTx for medical students, who are future clinicians who may need to discuss UTx as a treatment option for patients. As UTx becomes increasingly likely to be viable for transgender women in the future, there will need to be continued discussions on the ethics of performing the procedure and ensuring equitable access to women, especially in traditionally marginalized populations. Further discussion will also need to evaluate whether a clinician should be able to conscientiously object to UTx differently based on the gender identity of their patient given equal legality and clinical viability.

## Electronic supplementary material

Below is the link to the electronic supplementary material.

Supplementary Material 1

## Data Availability

The data that support the findings of this study are available from the corresponding author upon reasonable request.

## Abbreviations

UTx: Uterine transplant
OUWB: Oakland University William Beaumont School of Medicine
IQR: Interquartile range
SD: Standard deviation
