# Supplementary material for: Survey of medical student attitudes regarding uterine transplant for cisgender and transgender women: an observational study
Source: BMC Med Educ. 2025 Jul 6;25:1011. doi: 10.1186/s12909-025-07588-8 (PMC12232827; doi:10.1186/s12909-025-07588-8)
Supplement: Supplementary file 1 — Supplementary Material 1 [file 12909_2025_7588_MOESM1_ESM.docx]

Supplementary Appendix SA1

Survey instrument:

Uterine Transplantation Study

Start of Block: Information Sheet

Q1.1 A Survey of Medical Student Attitudes Towards Uterine Transplantation 
 You are being asked to participate in a research study conducted by Dr. Abram Brummett, Assistant Professor of Foundational Medical Studies at Oakland University William Beaumont School of Medicine. Your decision to participate in this study is voluntary. You can choose to stop your participation at any time or skip any part of the study if you are not comfortable. Your decision will not affect your present or future relationship with Oakland University, the researcher, the Department of Foundational Medical Studies, or Oakland University William Beaumont School of Medicine.
 **What is the purpose of this study?** The purpose of this study is to gather information about the attitudes of medical students toward uterine transplantation for both cisgender and transgender women.  
 **Who can participate in this study?** You are being asked to participate in the study because you are a medical student.   **Who is the financial sponsor for this study?** There is no financial sponsor for this study. Research team members have no conflicts related to this funding.
 **What do I have to do?** You will be asked to complete an anonymous online survey. The study will take about 10 minutes of your time to complete and can be done at a time and location of your choice.   **Are there any risks to me?** The survey has minimal risks, but some questions may cause discomfort. You can skip questions or end the survey anytime. Sharing opinions on sensitive topics may raise privacy concerns. You should be aware that your responses will be kept anonymous.   **Are there any benefits to me?** Although there may be no direct benefits to you, the results of this study may benefit others in the future.   **Will I receive anything for participating?** You will not receive anything for participating in this study.   **What if I want to stop participating in this study?** If you want to stop participating, close your browser before clicking ‘submit.’ If you click ‘submit,’ it will not be possible to stop participating.   **Who can I contact if I have questions about this study?**
 Dr. Abram Brummett, abrummett@oakland.edu, (248) 370-3618.    For questions regarding your rights as a participant in human subject research, you may contact the Oakland University Institutional Review Board at 248-370-4898.

End of Block: Information Sheet

Start of Block: Consent

Q2.1 Do you agree to participate?

- Yes (1)
- No (2)

Skip To: End of Survey If Do you agree to participate? = No

End of Block: Consent

Start of Block: Introduction

Q3.1 The first uterine transplantation in the U.S. occurred in 2016 at the Cleveland Clinic and is currently available for some cisgender women, such as those with uterine factor infertility, a previously irreversible form of infertility affecting hundreds of thousands of reproductive-aged women in the U.S.  The process involves a uterus from either a living or deceased donor being transplanted into the recipient, who is then placed on immunosuppression and artificially inseminated.  After giving birth via cesarean section, the uterus is removed by hysterectomy. Medical specialties involved include family medicine, internal medicine, OB/GYN, pediatrics, general surgery, and plastic surgery.  The cost of uterine transplantation is approximately $250,000, which covers the comprehensive package of IVF, pregnancy, and delivery.    Current criteria require recipients of uterine transplantations to be genetically female, but some have raised the possibility of uterine transplantation for transgender women. One Canadian research team has explored the clinical possibility of this option, concluding that there is “no scientific barrier to eventually providing transgender women with functional uterus transplants” (Jones et al, 2019).

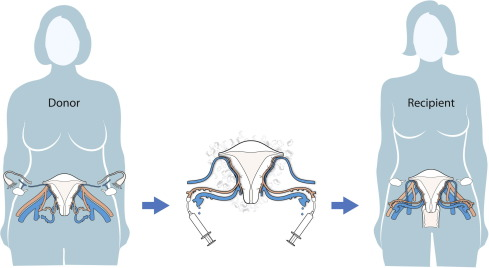


In what follows, you will be asked to provide anonymous responses regarding your attitudes and perspectives with respect to uterine transplantation for both cisgender and transgender women.

End of Block: Introduction

Start of Block: Demographic

| 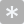 |
| --- |

Q4.1 What is your age in years?

________________________________________________________________

Q4.2 What is your gender?

- Male (1)
- Female (2)
- Transgender Male (3)
- Transgender Female (4)
- Non-binary / Third-gender (5)
- Prefer not to say (6)
- A gender not listed (write-in) (7) __________________________________________________

Q4.3 What is your political affiliation?

- Republican (1)
- Independent (2)
- Democrat (3)
- No Affiliation (4)
- Other (write in) (5) __________________________________________________

Q4.4 With what religious institution are you affiliated?

- Protestantism (1)
- Catholicism (2)
- Other Christian (3)
- Judaism (4)
- Islam (5)
- Buddhism (6)
- No affiliation (7)
- Other (write in) (8)

Q4.5 Please specify your ethnicity (Check all that apply)

- African American or Black (1)
- Asian (2)
- Caucasian or White (3)
- Indigenous or Native American (4)
- Latin or Hispanic (5)
- Middle Eastern or Northern African Descent (6)
- Native Hawaiian or Pacific Islander (7)
- Other (write-in) (8)

Q4.6 What medical specialty are you currently most interested in?

________________________________________________________________

End of Block: Demographic

Start of Block: Attitudes & Perspectives

The following questions ask about your perspectives on uterine transplantation for cisgender and transgender women.  When prompted indicate how strongly you agree or disagree with the statements for each population.

Q5.1 Uterine transplantation should be legal in the United States for:

|  | Strongly Disagree (1) | Disagree (2) | Agree (3) | Strongly agree (4) |
| --- | --- | --- | --- | --- |
| Cisgender Women (1) |  |  |  |  |
| Transgender Women (2) |  |  |  |  |

Q5.2 Please briefly explain your response for the question above.

________________________________________________________________

| Page Break |  |
| --- | --- |

Q6.1 If it is legalized, government funding should be used to support research on uterine transplantation for:

|  | Strongly Disagree (1) | Disagree (2) | Agree (3) | Strongly agree (4) |
| --- | --- | --- | --- | --- |
| Cisgender Women (1) |  |  |  |  |
| Transgender Women (2) |  |  |  |  |

Q6.2 Please briefly explain your response for the question above.

________________________________________________________________

| Page Break |  |
| --- | --- |

Q7.1 If it is legal and clinically viable, clinicians should be able to conscientiously object to providing, or assisting in the provision of, uterine transplantation for:

|  | Strongly Disagree (1) | Disagree (2) | Agree (3) | Strongly agree (4) |
| --- | --- | --- | --- | --- |
| Cisgender Women (1) |  |  |  |  |
| Transgender Women (2) |  |  |  |  |

Q7.2 Please briefly explain your response for the question above.

________________________________________________________________

| Page Break |  |
| --- | --- |

Q8.1 If it is legal, clinically viable, and clinicians are permitted to conscientiously object, you would conscientiously object to providing, or assisting in the provision of, uterine transplantation for:

|  | Strongly Disagree (1) | Disagree (2) | Agree (3) | Strongly agree (4) |
| --- | --- | --- | --- | --- |
| Cisgender Women (1) |  |  |  |  |
| Transgender Women (2) |  |  |  |  |

Q8.2 Please briefly explain your response for the question above.

________________________________________________________________

End of Block: Attitudes & Perspectives
